# The predictive role of olfactory identification on episodic memory and mild cognitive impairment: Results from the CIMA-Q cohort

**DOI:** 10.1177/13872877251378386

**Published:** 2025-09-12

**Authors:** Benoît Jobin, Natalie A Phillips, Johannes Frasnelli, Benjamin Boller, The CIMA-Q group

**Affiliations:** 1 Department of Psychology, 14847 Université du Québec à Trois-Rivières, Trois-Rivières, Quebec, Canada; 2 Research Centre of the Institut universitaire de gériatrie de Montréal, Montréal, Quebec, Canada; 3 Research Centre of the Hôpital du Sacré-Cœur de Montréal, Montréal, Quebec, Canada; 4 Department of Psychology, 5618 Concordia University, Montréal, Quebec, Canada; 5 Center for Research in Human Development (CRDH), 5618 Concordia University, Montréal, Quebec, Canada; 6 Centre for Research on Brain, Language & Music (CRBLM), 5620 McGill University, Montréal, Quebec, Canada; 7 Bloomfield Centre for Research in Aging, Lady Davis Institute for Medical Research/Jewish General Hospital/McGill University, Montréal, Quebec, Canada; 8 Department of Anatomy, Université du Québec à Trois-Rivières, Trois-Rivières, Quebec, Canada

**Keywords:** Alzheimer's disease, episodic memory, mild cognitive impairment, olfactory identification, subjective cognitive decline

## Abstract

**Background:**

Olfactory identification decline is a known early marker of Alzheimer's disease and is already present at the mild cognitive impairment (MCI) stage. While being linked with episodic memory, its predictive value for cognitive performance and distinguishing between clinical stages remains unclear.

**Objective:**

This study examined (1) the predictive value of olfactory identification for episodic memory performance and (2) its utility for discriminating individuals with MCI from those with subjective cognitive decline (SCD).

**Methods:**

Participants included 45 individuals with MCI (mean age = 80.08, SD = 5.86) and 48 with SCD (mean age = 75.82, SD = 5.64) from the Consortium for the Early Identification of Alzheimer's Disease–Quebec cohort. We evaluated olfactory identification with the University of Pennsylvania Smell Identification Test (UPSIT), and episodic memory with the Rey Auditory Verbal Learning Test (RAVLT). LASSO regression models were used to predict RAVLT total and delayed recall scores, using 80% of data for training and 20% for testing.

**Results:**

UPSIT significantly predicted both RAVLT total (β = 0.45, *p* = 0.03) and delayed recall (β = 0.18, *p* = 0.02), independent of diagnostic group. Including UPSIT in the models increased explained variance from 9% to 19% for total recall, and from 8% to 20% for delayed recall. The MCI group had significantly lower UPSIT performance than the SCD group (*p* = 0.01). Linear discriminant analysis yielded 69% classification accuracy, with higher specificity (79%) than sensitivity (58%).

**Conclusions:**

Olfactory identification enhances prediction of episodic memory performance and may be used as a cost-effective, non-invasive early screening tool for MCI.

## Introduction

Alzheimer's disease (AD) can be defined as a neurodegenerative disease characterized by a slow and progressive accumulation of amyloid-β depositions and neurofibrillary tau occurring over decades.^1–5^ These depositions initially accumulate in key structures as the hippocampus, parahippocampal gyrus, and entorhinal cortex, ultimately leading to cognitive impairments.^6–10^ Episodic memory responsible for the recall of personal experiences and events is typically the first cognitive domain to be impaired at the mild cognitive impairment (MCI) stage of the disease.^8,11–13^ In individuals with subjective cognitive decline (SCD), the stage preceding MCI where individuals perceive cognitive decline despite objective cognitive performance within normal limits,^14,15^ hippocampal volume was associated with episodic memory performance, which was not the case in cognitively healthy older adults.^16^

While SCD and MCI are risk factors for further cognitive decline and AD,^17–20^ these populations are heterogeneous, and not every individual converts to dementia or tests positive for tau and amyloid biomarkers. Approximately 12% of individuals under 75 with SCD progress to MCI or dementia, rising to 28% among those over 75.^21^ Positivity for AD biomarkers in SCD varies widely, from 10% to 76%.^22–24^ Annual conversion rates from MCI to AD dementia range from 7.5% to 16.5%, with about half testing negative for AD biomarkers.^25,26^ As treatments for AD become more available,^27–29^ the need to better target at-risk individuals increases. Identifying other early clinical markers alongside SCD and MCI will better characterize individuals at higher risk for AD and cognitive decline.

Olfactory decline is an early clinical marker of AD,^30,31^ especially involving odor identification and odor recognition impairments. This is in contrast to Parkinson's disease, which is characterized by a general olfactory impairment across different olfactory tasks.^32^ Individuals with AD exhibit a more specific impairment in their ability to identify and recognize odors;^32^ the same is true for individuals with MCI,^33,34^ with a trend toward lower olfactory identification in SCD.^35^ At the dementia and MCI stages of AD, olfactory processing areas in the medial temporal lobe (i.e., piriform cortex, entorhinal cortex, amygdala) are damaged,^36–41^ and olfactory identification scores have been associated with regional tau accumulation within the medial temporal lobe.^42–44^ Olfactory identification was associated with entorhinal cortex and hippocampal morphometry in patients with MCI^45–51^ and individuals with SCD without cognitive impairment.^52^

In this context, it is important to highlight that odor identification and episodic memory are associated in older adults without cognitive impairment,^53^ and both functions follow similar declining trajectories in aging.^54^ When compared, patients with amnestic MCI exhibited lower olfactory identification than those with non-amnestic MCI.^55^ A possible genetic underpinning is the ɛ4 allele of the Apolipoprotein E (*APOE*) gene: in carriers, long-term episodic memory decline and odor identification impairment are correlated, which was not the case in non-carriers.^56^

Olfactory testing could thus serve as a tool to screen for cognitive impairment in conditions such as preclinical AD.^57^ In this study, we therefore aimed to assess the predictive power of olfactory identification testing on episodic memory performance in individuals at risk for AD. More specifically, we aimed to (1a) assess the relationship between olfactory identification and episodic memory performance; (1b) assess the predictive value of olfactory identification for episodic memory functioning; (2a) compare olfactory identification performance between participants with MCI and those with SCD; and (2b) examine the discriminative value of olfactory identification to distinguish between MCI and SCD.

## Methods

The study was conducted in accordance with the Declaration of Helsinki and received approval from the Ethics Committee at the *Institut universitaire de gériatrie de Montréal*'s research center. All participants gave written consent after a detailed explanation of the study.

### Participants

Data for this study came from the Consortium for the Early Identification of Alzheimer's Disease-Quebec (CIMA-Q),^58^ launched in 2013 with initial funding from Fonds de recherche du Québec – Santé (FRQS) and Pfizer. The main objective of CIMA-Q is to build a thoroughly characterized cohort of older adults, incorporating clinical, cognitive, neuroimaging, and blood-based data. This resource is intended to (1) advance the early detection of AD, (2) offer a valuable cohort for use by researchers, (3) aid in the discovery of novel therapeutic targets aimed at preventing or delaying cognitive decline, and (4) support the development of future clinical research initiatives. The Institut universitaire de gériatrie de Montréal lead the CIMA-Q. The consortium represents a collaborative effort among Quebec researchers from Université Laval, McGill University, Université de Montréal, and Université de Sherbrooke.

Since 2014, CIMA-Q has recruited a longitudinal cohort of participants who are (1) cognitively normal, (2) with SCD, (3) with MCI, or (4) with Alzheimer's type dementia. In the current study, we included all eligible participants with SCD or MCI who underwent an olfactory examination between November 2022 and September 2024 (n = 93). All individuals included in the study were community-dwelling older adults aged 65 and above, living independently in the Canadian cities of Montreal, Sherbrooke, and Quebec City. Each participant completed an extensive neuropsychological assessment and was examined by expert physicians.

All recruitment procedures, clinical, cognitive, and neuropsychiatric measurements, and inclusion and exclusion criteria have been described previously.^58^ Participants from the MCI group (Montreal Cognitive Assessment, MoCA,^59^ score between 20 and 26) met the National Institute on Aging and the Alzheimer's Association (NIA-AA) clinical criteria for MCI:^11^ (1) self-reported cognitive decline; (2) measurable objective cognitive impairment, typically observed in episodic memory; (3) preserved functional independence in daily life; and (4) no diagnosis of dementia. Individuals in the SCD group, all of whom had a MoCA score of 26 or higher, met the diagnostic criteria established by the Subjective Cognitive Decline Initiative.^14^: (1) a self-reported cognitive decline, (2) normal range cognitive performance, (3) not meeting MCI criteria. Participants were excluded if they had significant neuropsychiatric comorbidities, neurological diseases (e.g., subdural hematoma, epilepsy, or non-Alzheimer's dementias), a history of intracranial surgery, active substance dependence, high-dose benzodiazepine use, or any condition deemed likely to interfere with cognitive performance or study participation.

### Design

The CIMA-Q study is a multicenter, large-scale longitudinal initiative that employs standardized cognitive and neuroimaging protocols across all participating sites (for additional information, visit http://www.cima-q.ca/). Initial eligibility was determined through a telephone-based pre-screening interview, which included the Telephone version of the Mini-Mental State Examination (t-MMSE),^60^ all eligible participants underwent a clinical examination and a neuropsychological assessment, which included an olfactory assessment.

### Episodic memory assessment

We used the Rey Auditory Verbal Learning Test (RAVLT) to assess episodic memory.^61^ In this task, a 15-item list of unrelated words was read to the participant across five trials, with the total number of recalled words recorded as the total recall score. Following an interference list, participants were asked to recall the original list immediately and again after a 30-min delay, the latter providing a delayed recall score.

### Olfactory identification assessment

The University of Pennsylvania Smell Identification Test (UPSIT) was used to measure olfactory identification.^62^ Participants were presented with a series of odorants embedded in scratch-and-sniff booklets and had to identify the odor from a set of four choice options. The test consists of 40 items, and the score represents the total number of correctly identified odors. Lower scores indicate poorer olfactory function, which may be associated with various neurological conditions.

### Statistical analysis

To analyze the association between the UPSIT and memory scores independently of diagnosis, we performed linear regression models. In each model, the RAVLT scores were the dependent variable, while diagnosis, age, sex, and education were included as covariates.

To analyze the predictive value of the UPSIT score on cognitive outcomes, we used the Least Absolute Shrinkage and Selection Operator (LASSO) regression models to identify significant predictors of cognitive outcomes. LASSO regression was chosen because it performs variable selection and regularization simultaneously, prevents overfitting and addresses multicollinearity. The target variables were RAVLT total and delayed recalls. For both scores, we computed two models. *Model 1* included age, sex, and education as predictors, while *Model 2* included age, sex, education, and UPSIT. The dataset was divided into training and testing subsets, with 80% allocated for model training and the remaining 20% set aside for evaluating model performance. To optimize the models, we performed 10-fold cross-validation and selected the optimal value of the regularization parameter (lambda, λ) based on the lowest mean squared error (MSE) from these cross-validation runs. For each model, we calculated the Root Mean Square Error (RMSE), R-squared (*R*²), and Pearson's *r* to assess the linear relationship between predicted and actual values. The analysis was repeated across 1000 iterations, with different random splits of the data, to ensure robust and stable estimates of model performance and coefficients.

To compare UPSIT scores between participants in the SCD and MCI groups, we performed an ANCOVA with UPSIT score as a within-subject factor and groups as a between-subject factor. Education, sex and age were used as covariates.

To assess the ability of UPSIT to discriminate SCD from MCI groups, we conducted a Linear Discriminant Analysis (LDA). The LDA model included UPSIT scores and demographic variables (age, sex, and education) as predictors to classify participants into the two diagnostic groups (SCD and MCI). Prior probabilities were calculated based on group proportions in the dataset, and the model estimated the group means for each predictor variable. The discriminant function coefficients were used to derive a linear combination of predictors that maximized the separation between the groups. Model performance was evaluated using overall classification accuracy, sensitivity (true positive rate for MCI), and specificity (true negative rate for SCD). We set the significance level (alpha) at 0.05 for all analyses.

## Results

Characteristics of participants are presented in Table 1.

**Table 1.** Characteristics of participants.

Variables	SCD n = 48 Mean (SD)	MCI n = 45 Mean (SD)	*p*
Age in years	75.82 (5.64)	80.08 (5.86)	**<0**.**001**
Female/Male	34/14	22/23	0.051
Years of Education	7.67 (4.20)	6.67 (4.02)	0.25
MoCA (/30)	27.81 (1.35)	23.51 (1.94)	**<0**.**001**
RAVLT total recall (/75)	50.04 (9.17)	40.91 (10.12)	**<0**.**001**
RAVLT delayed recall (/15)	10.85 (2.61)	7.32 (3.86)	**<0**.**001**
UPSIT (/40)	29.42 (6.13)	26.02 (6.49)	**0**.**01**

MCI: mild cognitive impairment; MoCA: Montreal Cognitive Assessment; RAVLT: Rey Auditory Verbal Learning Test; SCD: subjective cognitive decline; SD: standard deviation; UPSIT: The University of Pennsylvania Smell Identification Test.

Statistical T-test analyses indicated significant differences in age, cognitive, and olfactory measures (MoCA, RAVLT, UPSIT) between groups, with the MCI group exhibiting lower scores. No significant difference was found in sex distribution and years of education. RAVLT scores were missing for three participants. Bold indicates statistical significance (*p* < 0.05).

### Associations between the UPSIT score and episodic memory

The *UPSIT* was significantly and positively associated with the *RAVLT total recall* (*ß* = 0.45, *p* = 0.03). *Sex* (*ß* = −7.33 (lower scores in males), *p* < 0.001) was also significantly associated with *RAVLT total recall*, while *age* (*ß* = 0.03, *p* = 0.85) and *education* (*ß* = 0.25, p = 0.28) were not. The interaction term between *UPSIT* and *diagnosis* was not significant either (*ß* = 0.07, *p* = 0.80). The model accounted for 34% of the variance in the *RAVLT total recall* (*F* [6,83] = 8.84, *p* < 0.001, adj. *R*² = 0.35); three observations were missing and were therefore not included in the model.

**Figure 1.**
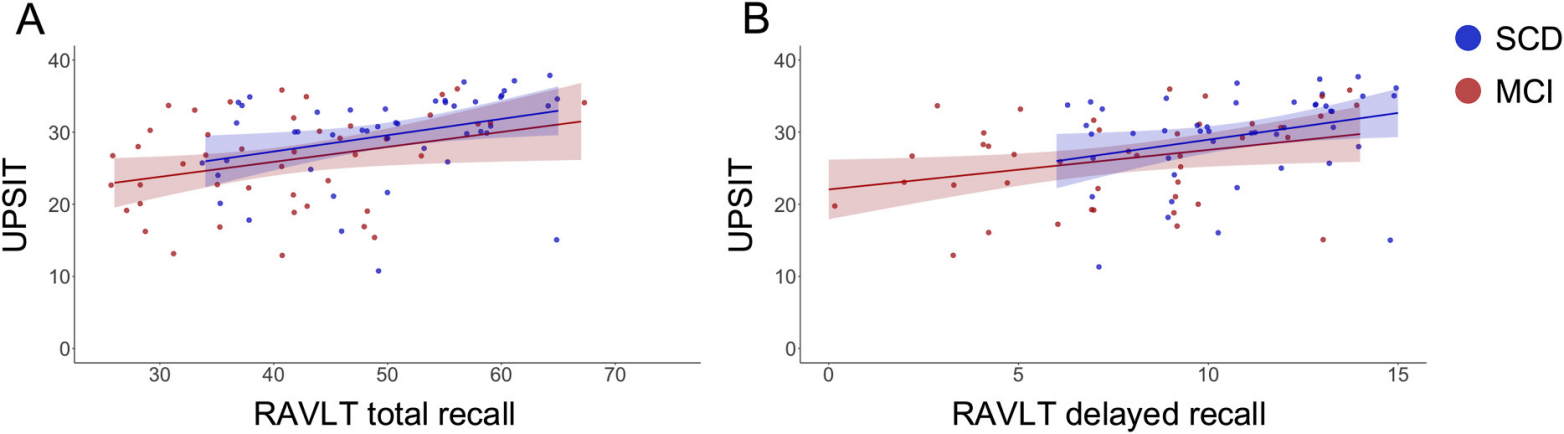
Associations between UPSIT and RAVLT total and delayed recall scores, adjusted for diagnosis, age, sex, and education. Each plot includes a regression line with a shaded 95% confidence interval. (A) UPSIT scores were positively associated with RAVLT total recall (*ß* = 0.45, *p* = 0.03). (B) UPSIT scores were positively associated with RAVLT delayed recall (*ß* = 0.18, *p* = 0.02).

Regarding delayed recall, the *UPSIT* was also significantly and positively associated with the *RAVLT delayed recall* (*ß* = 0.18, *p* = 0.02). *Sex* (*ß* = −2.06 (lower scores in males), *p* = 0.004) was also significantly associated with *RAVLT delayed recall*, while age (*ß* = 0.01, *p* = 0.84) and education (*ß* = 0.04, *p* = 0.66) were not. The interaction term between *UPSIT* and *diagnosis* remains non-significant (*ß* = −0.04, *p* = 0.68). The model accounted for 33% of the variance in the RAVLT delayed recall score (*F* [6,83] = 8.40, *p* < 0.001, adj. *R*² = 0.33); three observations were missing and not included in the model. (Figure 1)

### UPSIT score as a predictor of episodic memory

The LASSO regression models for each cognitive domain are reported in Table 2. *Model 1* explained 9% of the *RAVLT total recall* variance (RMSE = 10.41[CI: 10.35–10.48], adj. *R*² = 0.09 [CI: 0.08–0.09]), with *age* (*ß* = −0.39) and *education* (*ß* = 0.46) being significant predictors, while the coefficient for *sex* was not retained after regularization. When *UPSIT* was included in the model, *Model 2* explained 19% of the *RAVLT total recall* variance (RMSE = 9.83 [CI: 9.76–9.91], adj. *R*² = 0.19 [CI: 0.14–0.20]), with *age* (*ß* = −0.27), *education* (*ß* = 0.38), and *UPSIT* (*ß* = 0.55) being significant predictors, whereas *sex* remained non-contributory after regularization. The non-overlapping confidence intervals for adj. *R²* between *Models 1 and 2* suggest a meaningful contribution of olfactory identification to the models. RMSE (*t* = −22.45, *p* = <0.001) was significantly lower in Model 2, which included the UPSIT, with higher adj, *R*² (*t* = 20.89, *p* = <0.001).

**Table 2.** Results of LASSO regression models for predicting episodic memory scores.

Episodic memory scores	Predictors	*ß*	RMSE	*R* ^2^
	*Model 1*		10.41	0.09
			[10.35–10.48]	[0.08–0.09]
	Intercept	73.11		
	Age	−0.39		
	Sex	.		
	Education	0.46		
RAVLT total recall	*Model 2*		9.83	0.19
			[9.76–9.91]	[0.14–0.20]
	Intercept	48.90		
	Age	−0.27		
	Sex	.		
	Education	0.38		
	UPSIT	0.55		
	*Model 1*		3.72	0.08
			[3.69–3.75]	[0.07–0.09]
	Intercept	18.92		
	Age	−0.14		
	Sex	.		
	Education	0.11		
RAVLT delayed recall	*Model 2*		3.49	0.20
			[3.46–3.52]	[0.19–0.21]
	Intercept	11.41		
	Age	−0.10		
	Sex	.		
	Education	0.09		
	UPSIT	0.18		

For each RAVLT total and delayed recall score, two models were tested: Model 1 included age, sex, and education as predictors, while Model 2 added UPSIT score as an additional predictor. The table displays the mean metrics over 1000 iterations: standardized coefficients (β) for each predictor, Root Mean Square Error (RMSE), adjusted R-squared (R²). Dots (.) represent coefficients not retained in the model after regularization. Numbers in brackets represent the 95% confidence intervals.

Regarding the *RAVLT delayed recall*, *Model 1* explained 8% of the variance (RMSE = 3.72 [CI: 3.69–3.75], adj. *R*² = 0.08 [CI: 0.07–0.09]), with *age* (*ß* = −0.14) and *education* (*ß* = 0.11) being significant predictors, while the coefficient for *sex* was not retained after regularization. When *UPSIT* was included in the model, *Model 2* explained 20% of the variance (RMSE = 3.49 [CI: 3.46–3.52], adj. *R*² = 0.20 [CI: 0.19–0.21]), with *age* (*ß* = −0.10), education (*ß* = 0.09), and *UPSIT* (*ß* = 0.18) being significant predictors, whereas *sex* remained non-contributory after regularization. Again, the non-overlapping confidence intervals for adj. *R²* between *Models 1 and 2* suggest a meaningful contribution of olfactory identification to the models. RMSE (*t* = −21.02, *p* = <0.001) was significantly lower in Model 2, which included the UPSIT, with higher adj, *R*² (*t* = 18.78, *p* = <0.001).

### UPSIT score comparison between SCD and MCI groups

When comparing UPSIT scores between SCD and MCI groups, an ANCOVA revealed a significant effect of *group* demonstrating significantly lower scores in the MCI group compared to the SCD group (*F* [1,85] = 6.75; *p* = 0.01; *η^2^p* = 0.07). Main effects of *age* (*F* [1,85] = 2.06; *p* = 0.15; *η^2^p* = 0.02), *sex* (*F* [1,85] = 0.02; *p* = 0.89; *η^2^p* < 0.001), and *education* (*F* [1,85] = 0.57; *p* = 0.45; *η^2^p* < 0.001) were not significant, nor were interaction terms between *groups* and *age* (*F* [1,85] = 1.90; *p* = 0.17; *η^2^p* = 0.02), *groups* and *sex* (*F* [1,85] = 0.003; *p* = 0.96; *η^2^p* < 0.001), and *groups* and *education* (*F* [1,85] = 1.72; *p* = 0.19; *η^2^p* = 0.02).

### Group discrimination using UPSIT score

**Figure 2.**
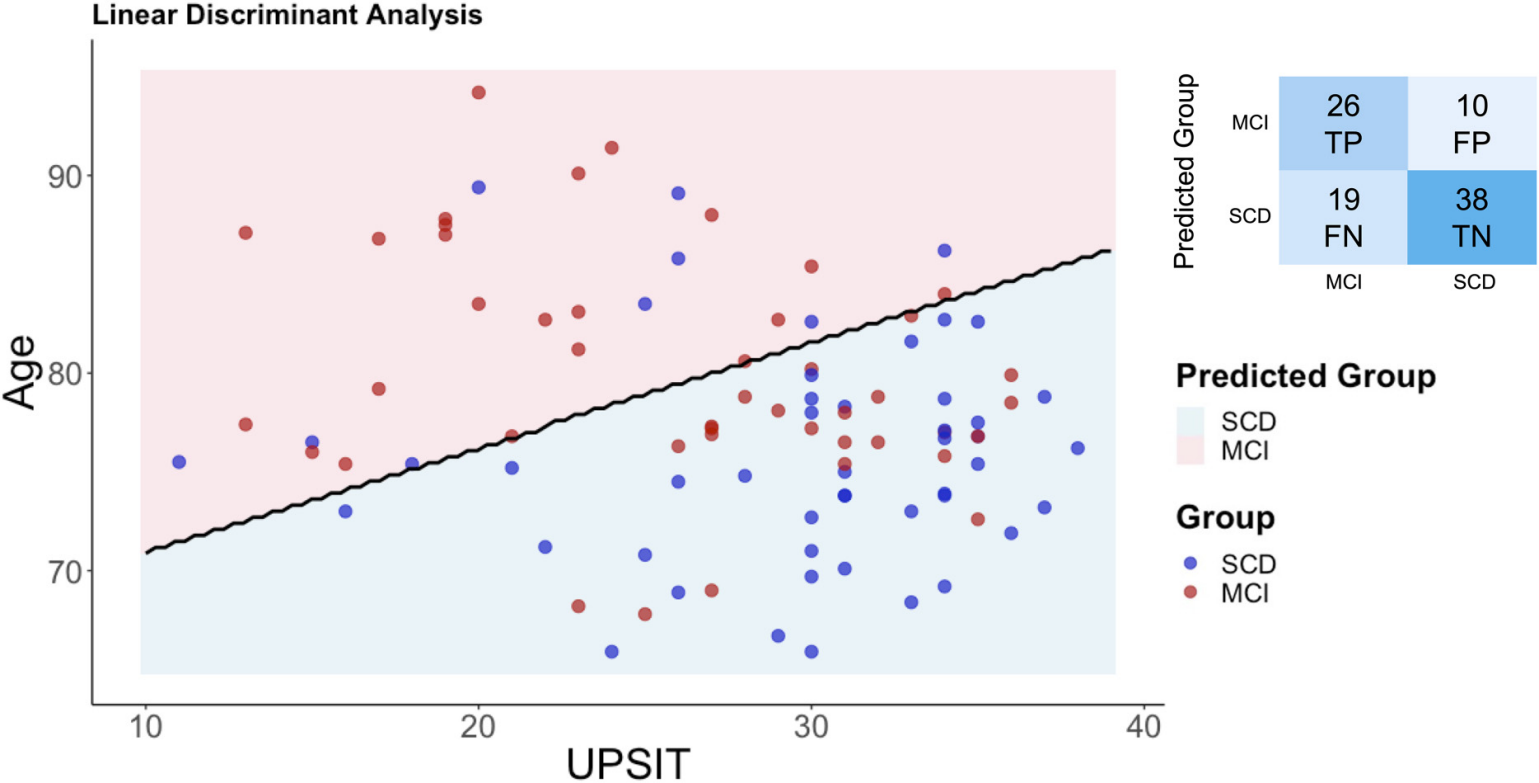
Linear discriminant analysis of UPSIT scores and age to differentiate between SCD and MCI groups. The scatter plot illustrates the distribution of SCD (blue dots) and MCI (red dots) participants based on UPSIT scores and age, with the black line representing the Linear Discriminant Analysis decision boundary. The shaded areas indicate predicted group classification: pink for MCI and blue for SCD. The confusion matrix summarizes classification results: true positives (TP), false positives (FP), false negatives (FN), and true negatives (TN). The model achieved 69% accuracy, with sensitivity (MCI detection) of 58% and specificity (SCD detection) of 79%.

LDA indicated that *UPSIT* scores (−0.066), *age* (0.125), *sex* (0.612), and *education* (−0.096) contributed to the discriminant function. The model classified participants into their respective groups with an overall accuracy of 69%. Sensitivity (true positive rate for MCI) was 58%, indicating that the model correctly identified 58% of individuals in the MCI group. Specificity (true negative rate for SCD) was 79%, reflecting a higher ability to classify SCD participants correctly. Among the 45 MCI participants, 26 were correctly classified as MCI (true positives), while 19 were misclassified as SCD (false negatives). Conversely, among the 48 SCD participants, 38 were correctly identified as SCD (true negatives), while 10 were misclassified as MCI (false positives). The decision boundary plot (Figure 2) demonstrates the separation between the SCD and MCI groups based on the predictors.

## Discussion

In this study, we assessed the predictive power of olfactory identification on episodic memory performance in individuals at risk for AD. We used a data-driven approach to investigate its predictive value on episodic memory performance and LDA to evaluate the utility of olfactory identification in distinguishing between SCD and MCI. Our findings revealed that olfactory identification was associated with RAVLT total recall and delayed recall scores, independently of diagnosis. Including UPSIT scores in predictive models significantly enhanced the explained variance from 9% to 19%, and from 8% to 20% for total recall scores and delayed recall scores, respectively. In terms of group comparisons, olfactory identification was significantly lower in individuals with MCI than in those with SCD. Using the olfactory identification score within LDA demonstrated moderate accuracy (69%) in distinguishing between SCD and MCI groups, with a lower sensitivity (58%) for detecting MCI than specificity for identifying SCD (79%). This indicates that the model was more effective at excluding MCI based on olfactory performance than identifying it within the CIMA-Q cohort.

We found significant associations between olfactory identification and episodic memory scores, independently of diagnosis. Thus, we demonstrated that olfactory identification correlated with episodic memory, replicating findings from the literature.^52^ Using a data-driven approach, we quantified the predictive value of olfactory identification scores on cognitive and episodic memory performance across all participants. LASSO models included UPSIT scores as significant predictors of RAVLT scores. When combined with demographic information from our participants, olfactory identification scores improved the explained variance for episodic memory scores. These results support the suggestion of using olfactory identification testing as an additional measurement for MCI screening^57^ and episodic memory performance in participants at risk for AD. Interestingly, episodic memory testing is the best predictor of AD dementia among cognitive measurements;^63^ with amnestic MCI patients being at higher risk of developing AD dementia compared to those with the non-amnestic presentation.^64,65^

The common cause hypothesis^66,67^ might explain the association between olfactory identification and episodic memory performance in participants at risk of AD, suggesting that the association between both functions results from underlying damage in common key areas (i.e., the hippocampus and entorhinal cortex).^6,7,68–72^ These brain regions are among the first damaged in AD pathology,^73^ and olfactory identification scores have been associated with cross-sectional^42,43^ and longitudinal tau accumulation in these central olfactory areas.^44^ Several studies have shown a relationship between olfactory identification and the morphometry of the hippocampus and entorhinal cortex in CN older adults,^46,74^ individuals with SCD,^52^ and patients with cognitive impairments associated with AD.^38,45,47–51^ Future studies will aim to validate this model of the common cause hypothesis, as well as the predictive role of olfactory measurements on medial-temporal lobe damages, by analyzing the shared variance explained by these damages on both episodic memory and olfactory decline, to elucidate the mechanisms underlying their relationship.

Our results replicated the literature on impaired olfactory identification in MCI.^33,34^ Furthermore, the LDA showed moderate accuracy (69%) in distinguishing between SCD and MCI groups, though its sensitivity for detecting MCI was lower than its specificity for identifying SCD. Clinically, among patients reporting cognitive decline, a higher olfactory identification score would be indicative of the absence of objective cognitive impairment (79%). In contrast, a lower olfactory score is less effective for screening MCI, with a sensitivity of 58%, indicating a need for further examination. These findings align with the understanding that while olfactory impairment is a recognized marker of AD,^30,75^ not all individuals with MCI progress to dementia, and only approximately half test positive for AD biomarkers.^25,26^ Furthermore, olfactory identification impairment is not exclusive to AD; reduced olfactory function can result from a variety of other conditions, including sinusitis, COVID-19, head trauma, and nasal polyps, among others, which is to consider when interpreting the result of this study and in general clinical settings.^76^ These considerations underscore the need for a comprehensive approach when interpreting olfactory identification scores in clinical and research contexts, recognizing their utility as part of a broader diagnostic framework rather than as a standalone diagnostic tool. To enhance sensitivity for detecting MCI, future research could investigate multimodal approaches that integrate olfactory testing with additional markers, such as cognitive performance, neuroimaging, or fluid biomarkers. Such integrative strategies may enhance early detection of neurodegenerative changes using smell tests as a first screening and improve clinical decision-making in individuals at risk.

Subjective Cognitive Decline Plus (SCD+) is a subtype of SCD characterized by the presence of additional risk factors associated with future cognitive decline. These include a self-reported decline specifically in memory, onset of symptoms within the past five years, age of 60 or older, personal concern regarding the decline, persistence of symptoms over time, medical-seeking consultation, and corroboration of the decline by someone close to the individual.^14,77^ These additional features aim to better characterize individuals with SCD as having an elevated risk of developing an objective cognitive decline. Olfactory identification is associated with AD biomarkers in the medial temporal lobe,^42–44^ long-term memory decline in *APOE* ε4 gene carriers,^56^ and the conversion to AD dementia stage.^78–80^ Future longitudinal studies should aim to assess the longitudinal prediction of episodic memory decline in individuals with SCD using olfactory identification scores.

This study has certain limitations. Although we cross-validated our predictive models by using an 80/20% split within our sample, the sample size was relatively small and drawn from a single cohort (CIMA-Q). Furthermore, the LDA was based on cross-sectional data. While our findings suggest that olfactory identification can moderately distinguish between SCD and MCI at a single time point, longitudinal studies are needed to determine the predictive value of odor identification for cognitive decline and disease progression over time. Also, participants were recruited from the community, suggesting that they may have had milder impairments and a lower likelihood of progressing to dementia than patients from memory clinics.^81,82^ It is possible that the observed effects could be even stronger in a memory clinic population. Replication of these results in larger, diverse, and independent samples, including clinical cohorts, would help to confirm their robustness and extend their generalizability.

To conclude, the results of this study showed that olfactory identification is specifically associated with episodic memory and enhances its prediction in individuals at risk of AD. LDA showed higher specificity (79%) for identifying SCD than sensitivity (58%) for detecting MCI. These findings highlight olfactory identification as a potential low-cost marker for cognitive screening, better suited to ruling out MCI than detecting it. Future studies should validate these results in larger, diverse samples and explore their integration with advanced biomarkers for AD.
